# Complete reversal of the clinical symptoms and image morphology of ketamine cystitis after intravesical hyaluronic acid instillation: A case report

**DOI:** 10.1097/MD.0000000000011500

**Published:** 2018-07-13

**Authors:** Ying-Lun Ou, Chin-Yu Liu, Tai-Lung Cha, Sheng-Tang Wu, Chih-Wei Tsao

**Affiliations:** a Division of Urology, Department of Surgery, Tri-Service General Hospital, National Defense Medical Center, Taipei; b Department of Nutritional Science, Fu Jen Catholic University, New Taipei City, Taiwan, Republic of China.

**Keywords:** bladder wall thickening, hyaluronic acid instillation, ketamine cystitis

## Abstract

**Rationale:**

Ketamine abuse is an emerging issue in many countries, and ketamine cystitis (KC) is a growing disease which more and more urologists may encounter with. There was no gold standard diagnostic criteria of ketamine cystits established yet, but well-accepted with the positive substance abuse history and clinical symptoms. The clinical presentation of ketamine cystitis varies and may mimic those presented in interstitial cystitis (IC), such as voiding frequency, urgency with urge incontinence, dysuria, nocturia, burning sensation during urination, post urination pain, painful hematuria, and small bladder capacity, but there are still differences that KC presented with more urgency, hematuria, pyuria and upper urinary tract involvement such as ureteral stenosis, vesico-ureteric reflux, hydronephrosis and renal function impairment.

**Patient concerns:**

We presented an interesting case with a 36-year-old man who's symptoms mimic acute prostatitis but there was no positive pathogen been cultured. The computed tomography (CT) findings revealed asymmetrical bladder wall thickening, which misleading us to the impression of bladder cancer. After the cystoscopy with bladder biopsy, the pathology revealed severe inflammation without malignancy. After that, we prescribed anticholinergic agent, beta-3 agonist and nonsteroidal anti-inflammatory drug (NSAID) for him, but in vain.

**Diagnoses:**

Erosive cystitis with prominent infiltration by eosinophils, lymphocytes, neutrophils and plasma cells.

**Interventions:**

Then we introduced hyaluronic acid (HA) instillation, once a week for total 10 times.

**Outcomes:**

After the treatment, his urgency, frequency, nocturia improved and his bladder capacity increased from less than 100ml to 350mL per urination. The following magnetic resonance imaging (MRI) and bladder biopsy result revealed complete reversal.

**Lessons:**

To our literature review, this is the first case of ketamine cystitis presented with asymmetrical bladder wall thickening, which may be considered as an irreversible change, but turns out complete reversal of the clinical symptoms and image morphology after merely intravesical hyaluronic acid instillation

## 1 Introduction

Ketamine is a phencyclidine derivative, which was first synthesized in the 1960 s as an antagonist of N-methyl-D-aspartic acid, and has been used as an anesthetic agent to date.^[1]^ However, it has also been abused as a recreational drug since the early 1990 s, especially among teenagers, as it exerts a variety of addictive effects.^[2,3]^ It is listed as a controlled drug/substance in many countries.

Ketamine cystitis (KC) is an emerging disease, first reported in 2007^[4]^; its presentation includes severe lower urinary tract symptoms (LUTS),^[5,6]^ such as increased voiding frequency, urgency with urge incontinence, dysuria, nocturia, a burning sensation during urination, post-urination pain, painful hematuria, and a small bladder capacity.^[7,8]^ In a study by Winstock et al,^[8]^ 1285 ketamine abusers were assessed, and 340 of them (26.6%) were found to suffer from LUTS. Another study performed in Taiwan revealed that 49.5% of the included ketamine abusers (1614 subjects were included in total) experienced LUTS.^[9]^ The severity of symptoms was found to be significantly correlated with the dose and frequency of use of ketamine.^[8]^ A recent study demonstrated that a history of ketamine use for at least 2 years, at a frequency of 3 or more times per week, may be associated with LUTS, and the symptoms may persist for 1 year after the cessation of drug use.^[10]^ Another study revealed that symptoms may present after 1 month of ketamine usage, and become more severe by the end of 1 year of use.^[7]^

## 2 Case report

A 36-year-old man who denied previous systemic disease had a history of drug abuse with ketamine for 6 to 7 years (at a frequency of 2–3 times per week, by nasal inhalation, and hence the dosage could not be measured), and had then ceased use for approximately 4 years.

He had suffered from dysuria, bladder pain, and a mild burning sensation during urination, especially over the urethral meatus and the perineal region, for approximately 1 month prior to admission. He ignored these symptoms initially, but the burning pain worsened, with concomitant urinary frequency and urgency. He visited another hospital for help, at which routine urine analysis revealed pyuria. Under the impression of acute prostatitis, oral antibiotic treatment with ciprofloxacin was initiated during an outpatient visit; however, his symptoms remained, with no improvement. Two days before admission to our hospital, the symptoms worsened, with a newly-developed decreased voiding amount (approximately 50 mL per void) and urgency with urge incontinence, accompanied by painful hematuria and blood clot formation, especially at the first urine void of the morning. The patient then presented to our Emergency Department. Urine analysis showed pyuria, over 100 white blood cells (WBCs)/high-power field (HPF), and significant tenderness and swelling of the prostate was noted upon digital rectal examination, but no pus-like urethral discharge was seen. Under the impression of acute prostatitis, for which oral antibiotic treatment had failed, the patient was then advised to undergo hospital admission for advanced antibiotic treatment.

**Figure 1:**
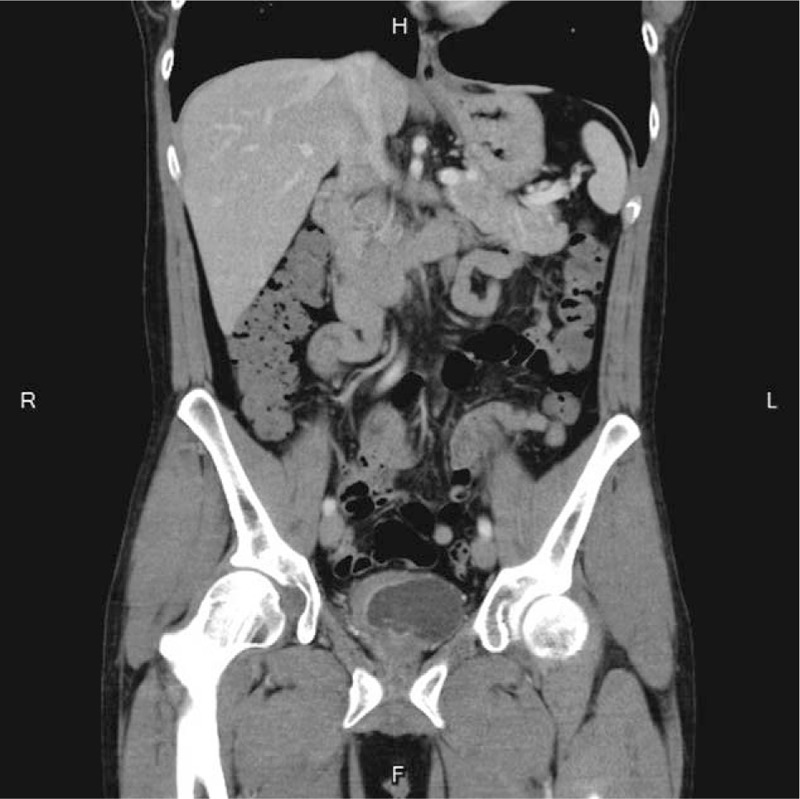
CT showed asymmetrical wall thickening (thickness of up to 1.2 cm) of the anterior aspect of the urinary bladder with mural nodules and mucosal enhancement with perivesical fatty stranding.

**Figure 2:**
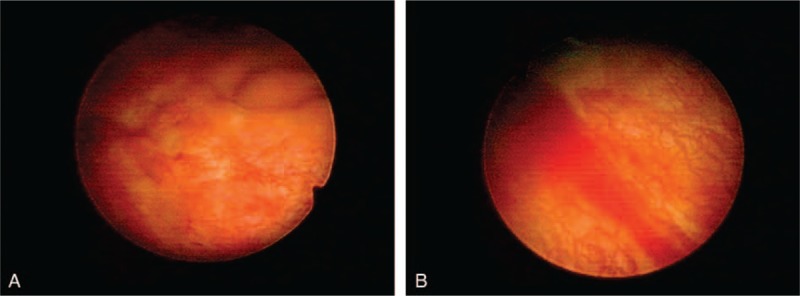
A, Irregular bladder mucosa surface with hump-like changes over the anterior aspect of the bladder wall without tumor lesions (location at which CT revealed one nodule). B, Easy bleeding after hydrodistension with glomerulation and ulcerative change.

**Figure 3:**
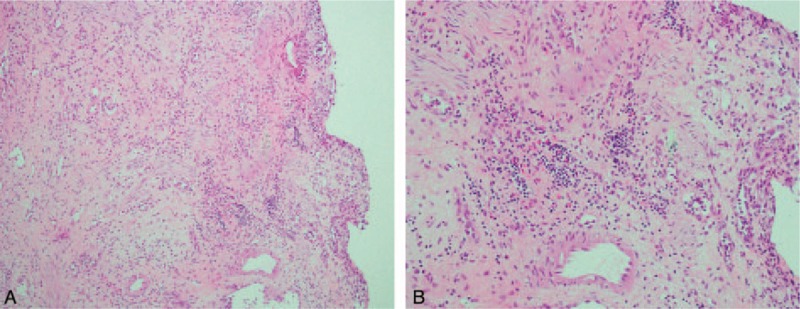
A, Bladder biopsy (100 ×). A denuded urothelium, infiltration of inflammatory cells, hypervascularity, and granulation tissue formation with fibrosis were seen. B, Bladder biopsy (200 ×). Infiltration by eosinophils, lymphocytes, neutrophils, and plasma cells was seen.

After admission, we consulted an infectious disease specialist for evaluation, and antibiotic treatment with ceftriaxone was started immediately. A blood test revealed WBC 4870/μL without predominance of neutrophils or eosinophils. No marked elevation of serum C-reactive protein (CRP) (0.25 mg/dL) was noted. The patient's temperature after admission had remained within the normal range, and there were no accompanying signs or symptoms of toxicity. Several blood and urine cultures were performed, including tuberculosis, but all results were negative. The symptoms of pyuria (which remained over 100 WBCs/HPF), urgency, and painful hematuria persisted with no improvement after one week of intravenous antibiotic treatment. However, a sonogram performed upon admission revealed suspected bladder wall thickening. Due to the persistent symptoms that failed to respond to advanced intravenous antibiotic treatment for 1 week, abdominal computed tomography (CT) with contrast was then arranged. The CT scan showed asymmetrical wall thickening (thickness of up to 1.2 cm) of the anterior aspect of the urinary bladder with a mural nodule, and mucosal enhancement with perivesical fatty stranding (Fig. 1). According to the above findings and the clinical symptoms, bladder cancer was highly suspected, and we discussed cystoscopy with bladder biopsy with the patient and his wife, which was then performed the next day. Prior to hydrodistension, the bladder mucosa presented with hypervascularity, but there was no tumor over the anterior wall of the bladder as seen on the CT scan. The bladder mucosa of the anterior wall was erythematous, with multiple hump-like changes, and several biopsies were performed. After hydrodistension at a pressure of 90 cm H_2_O for 8 minutes, the bladder capacity was approximately 150 mL, and bleeding over multiple aspects of the bladder was seen, with glomerulation and ulcerative changes (Fig. 2 A and B). Pathologic analysis of the bladder biopsies showed erosive cystitis, characterized by denuded urothelial cells, with prominent infiltration by eosinophils, lymphocytes, neutrophils, and plasma cells over the mucosa and submucosal layer. In addition, hypervascularity and submucosal granulation formation with fibrosis were observed (Fig. 3 A and B).

**Figure 4:**
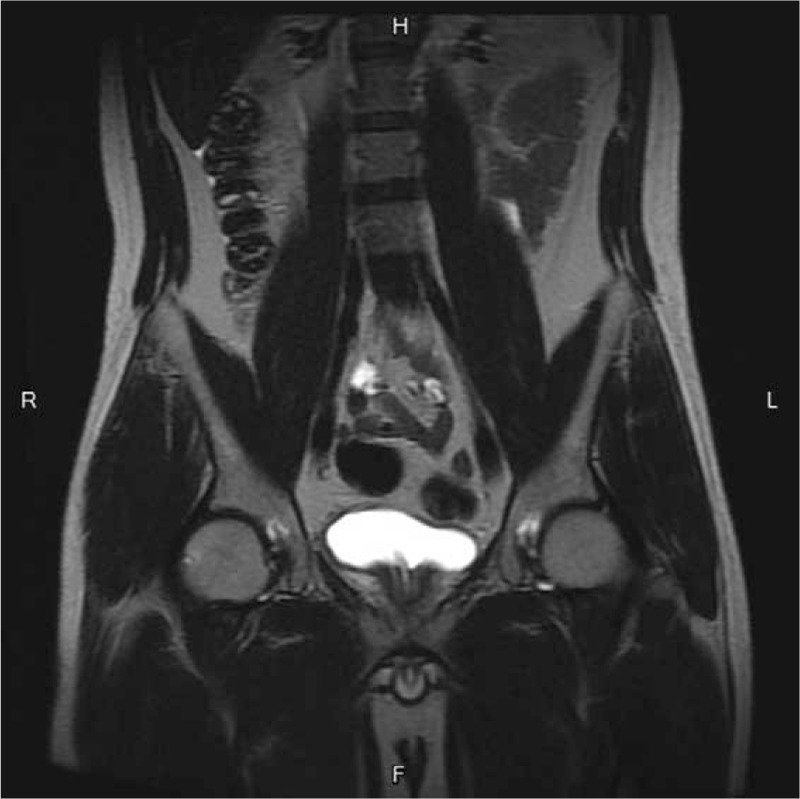
MRI of the bladder showed neither thickening of the bladder wall nor the existence of nodules.

**Figure 5:**
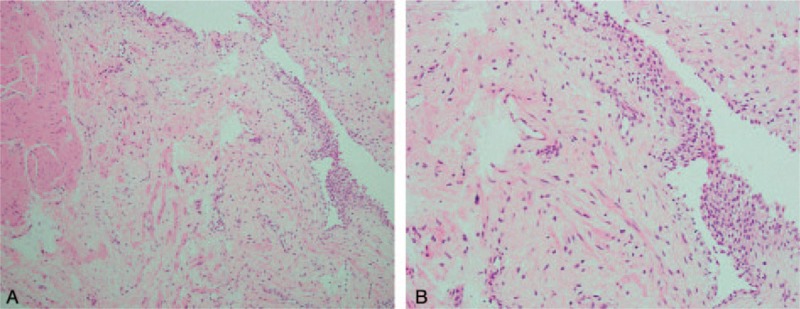
A, Bladder biopsy (100 ×). Regeneration of the urothelium was seen, with a lesser degree of infiltration of inflammatory cells. B, Bladder biopsy (200 ×). Lesser degree of infiltration of inflammatory cells.

After the operation, the bladder capacity increased a little, to approximately 70 to 80 mL per urination, but the urgency, frequency, nocturia, and hematuria still persisted. We also prescribed an anticholinergic agent, a beta-3 agonist and a nonsteroidal anti-inflammatory drug (NSAID), and the patient was then discharged. During 2 weeks of outpatient treatment, his symptoms did not improve with medication. Thus, we discontinued medical treatment and performed hyaluronic acid (HA) instillation, once a week for a total of 10 times. After the treatment, the symptoms of urgency, frequency and nocturia improved, and the bladder capacity increased to 350 mL per urination according to the patient's own voiding diary; in addition, no morning hematuria or hematuria after holding back urine occurred. After the patient's symptoms had improved, we arranged follow-up MRI of the bladder and cystoscopy; on the images, no thickening of the bladder wall nor nodules were observed (Fig. 4). Cystoscopy showed marked improvement of the previously-noted erythematous bladder mucosa, and there was neither active bleeding nor glomerulation seen during the whole procedure. Bladder biopsy near the previous biopsy site was performed, and the final pathologic analysis showed decreased inflammatory cell infiltration, regeneration of the urothelium, and less vascularity (Fig. 5 A and B).

Written informed consent to publish this case report was provided by the patient, and the consent procedure was approved by the Ethics Committee of Tri-Service General Hospital.

## 3 Discussion

This interesting case was treated as acute prostatitis at first by several urologists, including ourselves, and was then considered to be bladder cancer with irritative symptoms after failure of intravenous advanced antibiotic treatment and a CT scan. However, the final diagnosis was late-onset or acute exacerbation of KC. The exact pathophysiology of KC is still unclear, but the cornerstone of the process may be accumulation of ketamine and its metabolites, including nor-ketamine and hydroxyl-norketamine, which can be measured in high quantities in urine and induce an inflammatory or autoimmune response of the bladder wall, leading to ulcerative cystitis.^[11,12]^ Diagnosis of ketamine-related cystitis may depend on a history of substance abuse, clinical symptoms, cystoscopic findings, urine analysis, pathologic findings, and imaging modalities such as CT, sonography, and intravenous urography. There are no strict diagnostic criteria at present, but diagnosis can usually be made if the patient presents with a positive ketamine abuse history and typical clinical symptoms.

The typical clinical symptoms mentioned above may be accompanied by flank pain secondary to vesico-ureteral reflux (VUR), which is considered a profound complication of KC.^[13]^ The signs and symptoms of KC are similar to those of interstitial cystitis/ bladder pain syndrome

(IC/BPS), but differences do exist, in that KC presents with a greater urgency, hematuria, pyuria and upper urinary tract involvement such as ureteral stenosis, VUR, hydronephrosis, and renal function impairment.^[14]^ Urine analysis and urine culture show nonbacterial pyuria; cystoscopy may reveal erythematous change of the epithelium, mucosal tearing or laceration, neovascularization, and easy bleeding; and bladder glomerulation, petechial hemorrhages, and ulcer of the bladder mucosa may be present in severe cases, especially after bladder distension, which is similar to IC.^[12,15]^ The ureteroscopic findings of KC with hydronephrosis show swelling and edematous changes of the ureter mucosa, which imply inflammation extending to the ureter, and are rarely present in IC/BPS. In addition, the bladder wall usually presents with diffuse thickening in KC, while patients with IC/BPS usually present with a thin bladder wall.^[5,16]^ Renal sonography may reveal unilateral or bilateral hydronephrosis. Retrograde pyelography illuminates hydropnephrosis and a so-called “walking stick ureter,” characterized by segmental beading of ureteral strictures and straightening of the ureter.^[13]^ CT may reveal diffuse bladder wall thickening with peri-vesical fat inflammatory change. Also, ureteric wall thickening and dilatation may be present on CT images.^[15]^ Urodynamically, patients may present with either detrusor overactivity and/or decreased bladder compliance, and the functional bladder capacity is commonly < 150 mL.^[12]^ VUR has been detected to some degree, and was postulated to be a secondary event to severe bladder contraction with high detrusor pressure.^[7,17]^ Pathological changes include a denuded and focal reactive urothelium. The lamina propria shows granulation tissue formation and congested vessels. Mucosa and submucosal layers are infiltrated predominantly by lymphocytes, mast cells,epsinophils, and mast cells. Submucosal fibrosis, fibrinoid necrosis of arterioles and focal cacification, muscle hypertrophy, collagen accumulation, ureter wall thickening, and ureter mucosal infiltration by inflammatory cells and eosinophils is observed.^[5,7,12,14,18]^ The typical pathologic pattern of KC is consistent with eosinophil infiltration and muscle hypertorphy, differing from IC.^[5]^

According to our literature search, a few cases have presented with persistence of symptoms after ceasing usage of ketamine, and no case report has described late onset or acute exacerbation of KC as in the present case. One study noted that in approximately one-third of patients, symptoms are resolved; one-third continue to have persistent symptoms, and one-third experience worsening symptoms after cessation of ketamine abuse.^[8]^ Also, almost all studies describe the CT findings related to the bladder wall in KC as “generalized wall thickening,”^[5,15,19]^ but our patient presented with “asymmetrical wall thickening” with tumor-like lesions. It is well-known that some patients with bladder cancer present with LUTS, and a recent study revealed that a small proportion (4.1%) of patients with newly-diagnosed bladder cancer presented solely with LUTS,^[20]^ which misled us to an incorrect diagnosis.

A recent study including 27 patients with KC was conducted for evaluation of CT findings. The results were as follows: 1diffuse bladder wall thickening (24 patients, 88.9%); small bladder volume (18 patients, 66.7%); perivesical inflammation (12 patients, 44.4%) and hydronephrosis (12 patients, 44.4%); and ureteral wall thickening (9 patients, 33.3%).^[21]^ Other inflammatory and non-neoplastic bladder masses that may be accompanied by LUTS include pseudosarcomatous fibromixoid tumor, nephrogenic adenoma, malacoplakia, cystitis cystica, cystitis glandularis, eosinophilic cystitis, tuberculosis, and schistosomiasis, and so on.^[22]^ These can be excluded after pathological analysis.

The exact pathophysiology of KC is still unclear, but it may be related to: a high concentration of ketamine and its metabolites in the urine, with direct toxic effects on bladder interstitial cells, causing a submucosal inflammatory response; bladder barrier dysfunction; endothelial cell injury of microvessels; an autoimmune reaction of the bladder urothelium; neurogenic inflammation; urothelia atypia and carcinogenic gene overexpression; cell apoptosis; NOS- and cyclooxygenase-mediated inflammation.^[5,14]^

Various treatments have been used for KC, including antibiotics, NSAIDs, steroids, anticholinergics and hydrodistension medications, but all have failed to provide a significant and lasting improvement.^[7,12,14]^ Newly-developed beta-3 agonist Mirabegron was also prescribed to our patient, but still no significant improvement was seen. Beyond medication, intravesical instillation of a glycosaminoglycan (GAG), such as HA and chondroitin sulfate, has been demonstrated to improve symptoms in patients with IC/BPS,^[23]^ especially in terms of relieving bladder pain, frequency, and hematuria, but long-term follow-up is needed.^[7]^ GAG intravesical instillation may inhibit adherence of immune complexes, enhance connective tissue healing, reconstitute the defective GAG layer of the urothelium and improve its barrier function in IC/BPS patients.^[24,25]^ In 2 case series, investigators reported symptoms improvement in patients with KC who had received intravesical HA or oral pentosan polysulphate.^[26,27]^ Another case report illustrated one patient's successful response to treatment with chondroitin sulphate 0.2% over a 1-year period.^[28]^ Intravesical botulinum toxin serotype A (BoNT-A) injection may be an effective treatment, as it inhibits neurotransmitter release from nerve fibers, induces peripheral desensitization and improves neurogenic inflammation in patients with IC/BPS.^[29,30]^ It has also been proved to be effective in treating patients with KC, especially in terms of urinary frequency, nocturia, bladder pain, and bladder capacity 4 weeks after BoNT-A injection of 200U.^[31]^ However, due to the relative invasiveness of the procedure, some urologists only administer this treatment after failure of intravesical HA instillation.

Surgical intervention for KC may be indicated in patients with late-stage KC, characterized by bladder wall fibrosis, contracture, hydronephrosis, and possibly accompanied by renal function impairment.^[12]^ One study suggested that KC patients with a cystometric bladder capacity of less than 100 mL and low bladder compliance should undergo supratrigone cystectomy with augmentation enterocytoplasty or cystectomy with urinary diversion to preserve renal function.^[6]^ In our case, the CT finding of an asymmetrical wall thickness may be generally considered as an “irreversible change,” but this was improved merely by intravesical HA instillation.

A limitation of our case report was that no precise diagnostic criteria of KC have been established yet, and no case been reported with a pattern of acute exacerbation or late-onset KC after cessation of the use of ketamine for years, as in our patient. Why the clinical symptoms presented after years of cessation of using ketamine remains unclear, but it may be triggered by infection, such as acute prostatitis, or an autoimmune response to an unknown antigen, and hence further study is required.

## 4 Conclusion

The exact pathophysiology of KC requires further exploration, and the clinical presentation, including image findings, may vary in patients. We should keep in mind that a drug abuse history of ketamine, especially in the younger population, should always be taken. Before considering invasive treatment such as intravesical BoNT-A injection or bladder augmentation surgery, intravesical HA instillation may be a relatively safe and effective treatment modality. Our patient presented with irregular bladder wall thickening, which is considered an irreversible change; however, complete reversal was achieved after intravesical HA instillation.

## Author contributions

**Conceptualization:** Tai-Lung Cha.

**Data curation:** Chin-Yu Liu, Sheng-Tang Wu.

**Supervision:** Sheng-Tang Wu, Chih-Wei Tsao.

**Writing – original draft:** Ying-Lun Ou.

**Writing – review & editing:** Chih-Wei Tsao.

## References

[1] Corssen G Domino EF Dissociative anesthesia: further pharmacologic studies and first clinical experience with the phencyclidine derivative Cl-581. Anesth Analg 1966;45:29–40. 5325977

[2] Dillon P Copeland J Jansen K Patterns of use and harms associated with non-medical ketamine use. Drug Alcohol Depend 2003;69:23–8. 12536063 10.1016/s0376-8716(02)00243-0

[3] Smith KM Larive LL Romanelli F Club drugs: methylenedioxy-methamphetamine, flunitrazepam, ketamine hydrochloride, and gamma-hydroxybutyrate. Am J Health Syst Pharm 2002;59:1067–76. 12063892 10.1093/ajhp/59.11.1067

[4] Chu PS Kwok S Lam K Street ketamine’-associated bladder dysfunction: a report of ten cases. Hong Kong Med J 2007;13:311. 17592176

[5] Jhang JF Hsu YH Kuo HC Possible pathophysiology of ketamine-related cystitis and associated treatment strategies. Int J Urol 2015;22:816–25. 26087832 10.1111/iju.12841

[6] Chung SD Wang CC Kuo HC Augmentation enterocystoplasty is effective in relieving refractory ketamine-related bladder pain. Neurourol Urodyn 2014;33:1207–11. 23996856 10.1002/nau.22477

[7] Tsai TH Cha TL Lin CM Ketamine-associated bladder dysfunction. Int J Urol 2009;16:826–9. 19659678 10.1111/j.1442-2042.2009.02361.x

[8] Winstock AR Mitcheson L Gillatt DA The prevalence and natural history of urinary symptoms among recreational ketamine users. BJU Int 2012;110:1762–6. 22416998 10.1111/j.1464-410X.2012.11028.x

[9] Chen WY Huang MC Lin SK Gender differences in subjective discontinuation symptoms associated with ketamine use. Subst Abuse Treat Prev Policy 2014;9:39. 25245125 10.1186/1747-597X-9-39 PMC4183767

[10] Mak S Chan M Bower W Lower urinary tract changes in young adults using ketamine. J Urol 2011;186:610–4. 21684556 10.1016/j.juro.2011.03.108

[11] Moore KA Sklerov J Levine B Urine concentrations of ketamine and norketamine following illegal consumption. J Anal Toxicol 2001;25:583–8. 11599604 10.1093/jat/25.7.583

[12] Chu PSK Ma WK Wong SCW The destruction of the lower urinary tract by ketamine abuse: a new syndrome? BJU Int 2008;102:1616–22. 18680495 10.1111/j.1464-410X.2008.07920.x

[13] Huang PW Meng E Cha TL ‘Walking-stick ureters’ in ketamine abuse. Kidney Int 2011;80:895. 21960173 10.1038/ki.2011.242

[14] Meng E Wu ST Cha TL A murderer of young bladders: ketamine-associated cystitis. Urol SciV 24 2013;113–6.

[15] Lee CL Jiang YH Kuo HC Increased apoptosis and suburothelial inflammation in patients with ketamine-related cystitis: a comparison with non-ulcerative interstitial cystitis and controls. BJU Int 2013;112:1156–62. 23937072 10.1111/bju.12256

[16] Mason K Cottrell AM Corrigan AG Ketamine-associated lower urinary tract destruction: a new radiological challenge. Clin Radiol 2010;65:795–800. 20797465 10.1016/j.crad.2010.05.003

[17] Shahani R Streutker C Dickson B Ketamine-associated ulcerative cystitis: a new clinical entity. Urology 2007;69:810–2. 17482909 10.1016/j.urology.2007.01.038

[18] Wei Y Yang J Yin Z Genitourinary toxicity of ketamine. Hong Kong Med J 2013;19:341–8. 23832948 10.12809/hkmj134013

[19] Yek J Sundaram P Aydin H The clinical presentation and diagnosis of ketamine-associated urinary tract dysfunction in Singapore. Singapore Med J 2015;56:660–4. 26702160 10.11622/smedj.2015185 PMC4678404

[20] Dobbs RW Hugar LA Revenig LM Incidence and clinical characteristics of lower urinary tract symptoms as a presenting symptom for patients with newly diagnosed bladder cancer. Int Braz J Urol 2014;40:198–203. 24856486 10.1590/S1677-5538.IBJU.2014.02.09

[21] Huang LK Wang JH Shen SH Evaluation of the extent of ketamine-induced uropathy: the role of CT urography. Postgrad Med J 2014;90:185–90. 24443558 10.1136/postgradmedj-2013-131776 PMC3963547

[22] Wong-You-Cheong JJ Woodward PJ Manning MA Inflammatory and nonneoplastic bladder masses: radiologic-pathologic correlation. Radiographics 2006;26:1847–68. 17102055 10.1148/rg.266065126

[23] Giberti C Gallo F Cortese P Combined intravesical sodium hyaluronate/chondroitin sulfate therapy for interstitial cystitis/bladder pain syndrome: a prospective study. Ther Adv Urol 2013;5:175–9. 23904856 10.1177/1756287213490052 PMC3721440

[24] Sato H Takahashi T Ide H Antioxidant activity of synovial fluid, hyaluronic acid, and two subcomponents of hyaluronic acid. Synovial fluid scavenging effect is enhanced in rheumatoid arthritis patients. Arthritis Rheum 1988;31:63–71. 3345232 10.1002/art.1780310110

[25] Abatangelo G Martelli M Vecchia P Healing of hyaluronic acid-enriched wounds: histological observations. J Surg Res 1983;35:410–6. 6632867 10.1016/0022-4804(83)90030-6

[26] Chen C-H Lee M-H Chen Y-C Ketamine-snorting associated cystitis. J Formos Med Assoc 2011;110:787–91. 22248834 10.1016/j.jfma.2011.11.010

[27] Lai Y Wu S Ni L Ketamine-associated urinary tract dysfunction: an underrecognized clinical entity. Urol Int 2012;89:93–6. 22710265 10.1159/000338098

[28] Smart C Kabir M Pati J Treatment of ketamine-associated cystitis with chondroitin sulphate. Br J Nurs 2013;22. 10.12968/bjon.2013.22.Sup18.S4 24121772

[29] Chuang YC Yoshimura N Huang CC Intravesical botulinum toxin a administration produces analgesia against acetic acid induced bladder pain responses in rats. J Urol 2004;172:1529–32. 15371885 10.1097/01.ju.0000137844.77524.97

[30] Liu H-T Kuo H-C Intravesical botulinum toxin A injections plus hydrodistension can reduce nerve growth factor production and control bladder pain in interstitial cystitis. Urology 2007;70:463–8. 17905097 10.1016/j.urology.2007.04.038

[31] Shaojun J Keji X Yuebin C Treatment ketamine-related bladder dysfunction by intravesical injection of botulinum toxin A. J Third Mil Med Univ 2012;11:032.

